# Immunoglobulin G4‐positive interstitial pneumonia associated with pleuroparenchymal fibroelastosis

**DOI:** 10.1002/rcr2.925

**Published:** 2022-03-08

**Authors:** Keishi Sugino, Hirotaka Ono, Mikako Saito, Seiji Igarashi, Atsuko Kurosaki, Eiyasu Tsuboi

**Affiliations:** ^1^ Department of Respiratory Medicine Tsuboi Hospital Koriyama City Japan; ^2^ Department of Diagnostic Pathology Tsuboi Hospital Koriyama City Japan; ^3^ Department of Diagnostic Radiology Fukujuji Hospital, Anti‐tuberculosis Association Kiyose City Japan

**Keywords:** immunoglobulin G4, immunoglobulin G4‐related disease, interstitial pneumonia, pleuroparenchymal fibroelastosis

## Abstract

A 79‐year‐old former smoking Japanese man was admitted to our hospital with a 2‐year history of dry cough and dyspnoea on exertion. High‐resolution computed tomography of the chest revealed reticulation and perilobular opacity with bronchial wall thickening and ground‐glass opacities (GGOs) in both lungs, in addition to subpleural dense consolidation (pleuroparenchymal fibroelastosis‐like lesion; PPFE‐like lesion) predominantly in the bilateral upper lobes. Serum immunoglobulin G4 (IgG4) was elevated (348 mg/dl). Lung biopsy specimens obtained by video‐assisted surgery revealed a mixture of usual interstitial pneumonia (IP) and non‐specific IP pattern admixed with PPFE. In addition, immunohistochemical staining of IgG4 showed numerous IgG4‐positive plasma cells. Consequently, he was diagnosed with IgG4‐positive IP associated with PPFE. We initiated a combination therapy with prednisolone and cyclosporine as a calcineurin inhibitor. During prednisolone tapering, his clinical conditions and GGOs improved gradually over 12 months. However, reticular opacities and PPFE‐like lesions remained unchanged, and pulmonary function test findings slightly deteriorated.

## Graphical abstract

We describe a case of immunoglobulin G4‐positive interstitial pneumonia associated with pleuroparenchymal fibroelastosis, confirmed by video‐assisted thoracoscopic surgery. Combination therapy with prednisolone and cyclosporine showed long‐term efficacy and safety.

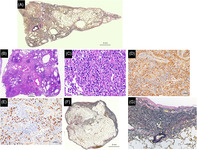

## INTRODUCTION

Immunoglobulin G4‐related disease (IgG4‐RD) is a recognized systemic fibro‐inflammatory disease characterized by elevated serum IgG4 concentrations, infiltration of IgG4‐positive plasma cells and fibrosis, which affects various organs including the lungs.^1^ Although IgG4‐related lung disease (IgG4‐RLD) is known to occur in approximately 10% of IgG4‐RD, whether condition such as IgG4‐positive plasma cells infiltration in the lungs without the extrathoracic lesions represents one of subtype of IgG4‐RD remains controversial.^2^ Moreover, there have been a few reports on IgG4‐related interstitial pneumonia (IP).^2^, ^3^, ^4^, ^5^ In addition, there is little information on clinical and histopathological characteristics and response to anti‐inflammatory drugs. We describe a case of IgG4‐positive IP associated with pleuroparenchymal fibroelastosis (PPFE), confirmed by video‐assisted thoracoscopic surgery (VATS). Combination therapy with prednisolone and cyclosporine showed long‐term efficacy and safety.

## CASE REPORT

**FIGURE 1:**
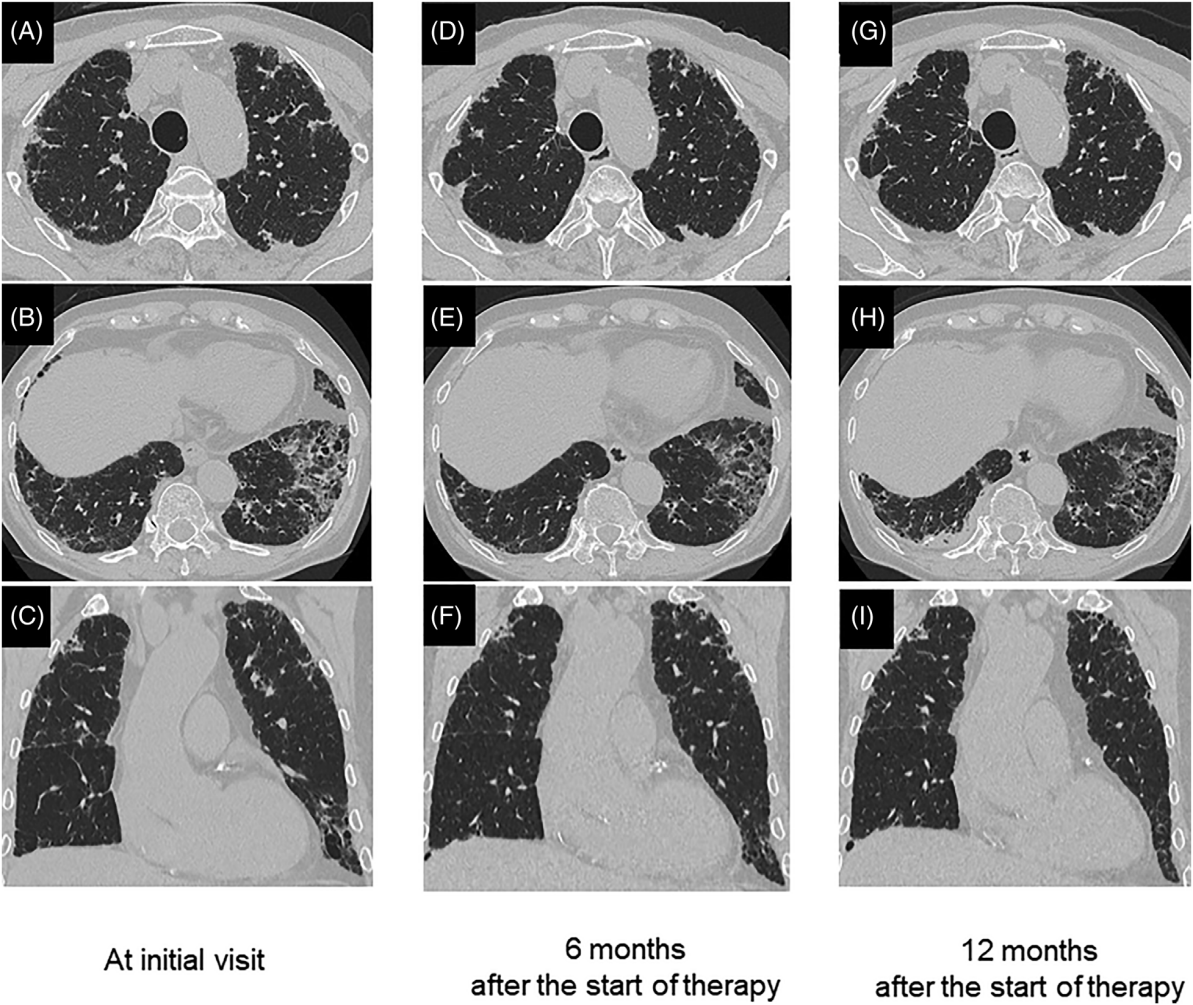
Serial changes in high‐resolution computed tomography (HRCT) of the chest. (A, B) At the initial visit, HRCT revealed reticulation and perilobular opacity with bronchial wall thickening and patchy ground‐glass opacities (GGOs) in the bilateral lower lobes, in addition to subpleural dense consolidation (pleuroparenchymal fibroelastosis‐like lesion; PPFE‐like lesion) predominantly in the bilateral upper lobes. (C) Coronal section of the chest HRCT showed dense subpleural consolidation and irregular septal thickening in the bilateral upper lobes predominance. (D–F) Six months after a combination therapy with cyclosporine and prednisolone began; interlobular septal thickening with bronchial wall thickening and GGOs in both lung fields were improved. PPFE‐like lesions remained unchanged. (G–I) Twelve months after the start of therapy, reticulation, thin‐walled bronchiectasis and PPFE‐like lesions remained unchanged

**FIGURE 2:**
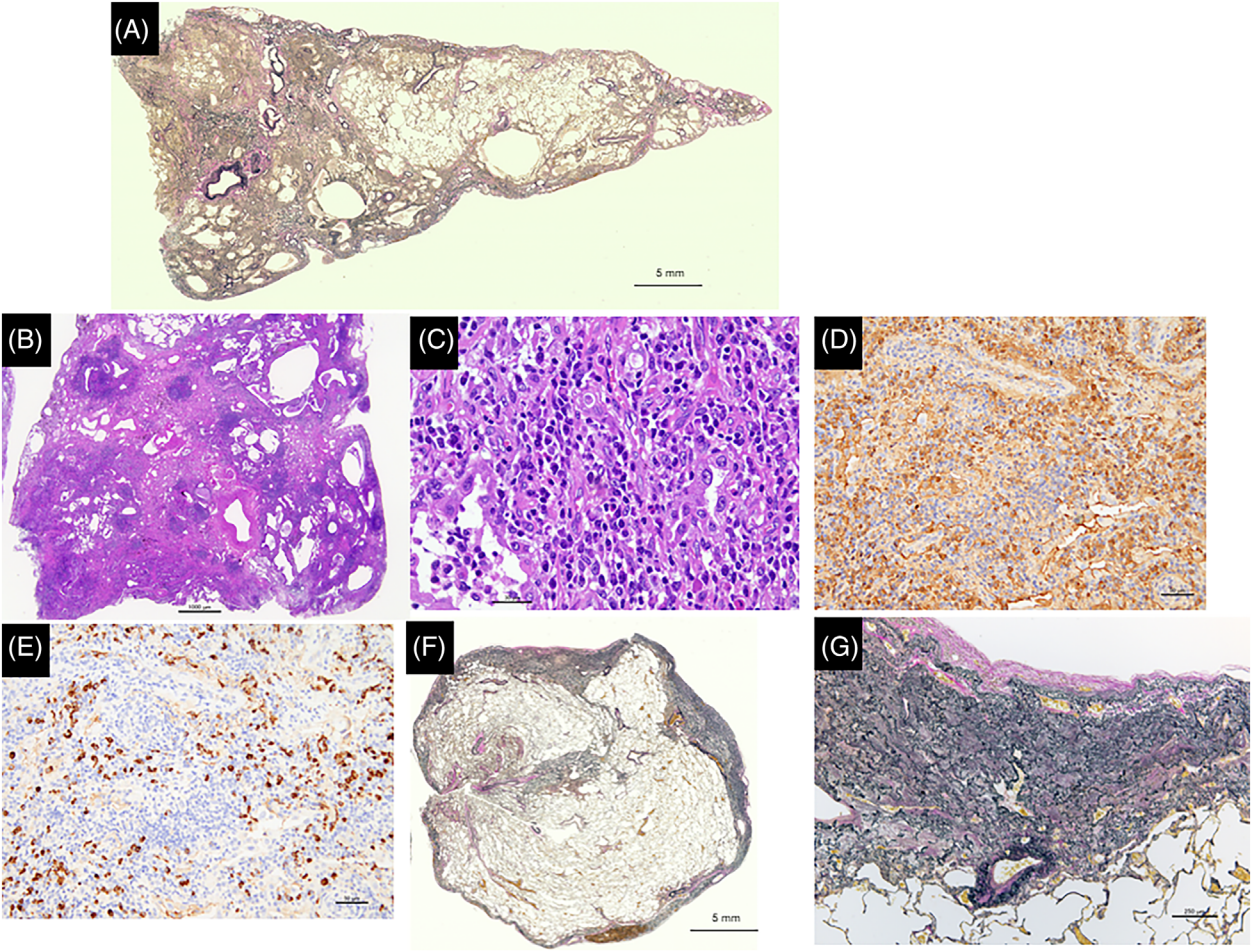
Histopathological findings of lung biopsy specimens obtained by video‐assisted thoracoscopic surgery. (A) Low‐magnification microscopic appearance of the right lower lobe revealed unclassifiable interstitial pneumonia (IP) composed of dominantly usual IP and non‐specific IP pattern (Elastica van Gieson stain; bar represents 5 mm). (B) There were scattered hyperplasia of lymphoid follicles in the fibrosis (haematoxylin–eosin stain; bar represents 1 mm). (C) Note the numerous lymphoplasmacytic cells infiltration in interlobular and peribronchial interstitium, and alveolar walls (haematoxylin–eosin stain; bar represents 30 μm) (D) Immunohistochemical staining of immunoglobulin G (IgG; bar represents 50 μm). (E) Immunohistochemical staining of IgG4 (bar represents 50 μm) revealed many IgG4‐positive plasma cells; the ratio of IgG4‐positive cells to IgG‐positive cells exceeded 50%. (F) Low magnified microscopic appearance of the right upper lobe revealed thickened fibrous pleura and subpleural fibroelastosis (Elastica van Gieson stain; bar represents 5 mm). (G) At higher magnification, intra‐alveolar fibrosis and interstitial elastosis were seen (Elastica van Gieson stain; bar represents 250 μm)

A 79‐year‐old former smoking Japanese man was admitted to our hospital with a 2‐year history of dry cough and dyspnoea on exertion. He had no history of recurrent lung infections such as non‐tuberculous mycobacterial infection. There was no evidence of specific physical findings suggestive of autoimmune diseases. High‐resolution computed tomography (HRCT) of the chest revealed reticulation and perilobular opacity with bronchial wall thickening and patchy ground‐glass opacities (GGOs) in the bilateral lower lobes, in addition to subpleural dense consolidation (PPFE‐like lesion) predominantly in the bilateral upper lobes (Figure 1A–C). Laboratory data showed high serum levels of a Krebs von den Lungen‐6 (KL‐6, 1065 U/ml) and a surfactant protein‐D (SP‐D, 313 ng/ml). Moreover, all tests of autoimmune antibodies yielded negative results. Arterial blood gas analysis revealed a pH of 7.46, partial pressure of carbon dioxide of 31.9 mm Hg and partial pressure of oxygen of 76.0 mm Hg in room air. Additional serological studies demonstrated elevated levels of IgG4 (348 mg/dl). The pulmonary function test showed normal range (forced vital capacity [FVC] of 2.66 L and 82.4% of predicted, forced expiratory volume in 1 s [FEV_1_] of 2.02 L and 79.5% of predicted) with diffusion capacity for carbon monoxide (DL_CO_ of 19.3 ml/min/mm Hg, 134.8% of predicted). Examination of bronchoalveolar lavage fluid (BALF) revealed alveolar macrophages, 84.6%; lymphocytes, 14.0%; neutrophils, 0.4%; and eosinophils, 0%, with increased total cells and a CD4/CD8 ratio of 3.0. Cultures of sputum and BALF were negative for fungal, bacterial or mycobacterial pathogens. Lung biopsy specimens obtained by VATS revealed dominantly usual IP and non‐specific IP (NSIP) pattern in the lower lobe, in addition to PPFE lesions in the upper lobe (Figure 2A,F,G). There were scattered hyperplasia of lymphoid follicles in the fibrosis and marked lymphoplasmacytic cells infiltration in interlobular and peribronchial interstitium, and alveolar walls (Figure 2B,C), but no evidence of obliterative phlebitis or arteritis, storiform fibrosis or neoplastic cells. In addition, immunohistochemical staining of IgG4 showed numerous IgG4‐positive plasma cells (Figure 2D,E). Consequently, he was diagnosed with IgG4‐positive IP associated with PPFE. We initiated a combination therapy with cyclosporine (150 mg/day) as a calcineurin inhibitor and prednisolone (30 mg/day). During prednisolone tapering, his clinical conditions and GGOs improved gradually over 6 months (Figure 1D–F). However, reticular opacities and PPFE‐like lesions remained unchanged at 12 months (Figure 1G–I), and values of FVC and DL_CO_ slightly deteriorated (FVC of 2.50 L and 77.2% of predicted, DL_CO_ of 18.4 ml/min/mm Hg, 127.1% of predicted). On the other hand, the serum IgG4 level decreased from 348 to 49 mg/dl. Also, serum KL‐6 and SP‐D decreased from 1065 to 579 U/ml and from 313 to 107 ng/ml, respectively. The patient had no relapse or adverse effects in the subsequent 12 months.

## DISCUSSION

We described a case of IgG4‐positive IP associated with PPFE, diagnosed histopathologically by VATS. Tanaka et al. have already described a case of IgG4‐positive IP mimicking idiopathic cellular NSIP without other IgG4 organ involvement.^3^ To date, many patients with IgG4‐RLD manifesting as IP have been discovered after involvement of other organs. Kono et al.^4^ also reported a case of IgG4‐RD associated with autoimmune pancreatitis and combined pulmonary fibrosis and emphysema.

As described by Ikeda et al.,^2^ IP with IgG4‐positive plasma cells without extrathoracic lesions may differ from previously proposed IgG4‐RLD, because there was no evidence of the following histological features: (i) storiform fibrosis and (ii) obliterative phlebitis or obliterative arteritis, which are known as important histological findings of IgG4‐RLD. More recently, Komatsu et al.^5^ defined IP with abundant IgG4‐positive cells infiltration and elevated serum IgG4 levels without extrathoracic lesions as IgG4‐positive IP and studied clinico‐radio‐pathological characteristics of this IP. As a result, none of these patients had either storiform fibrosis and obstructive vasculitis. Only one of 16 patients had histologically PPFE lesions as seen in our patient. Moreover, although GGOs on chest HRCT improved for all patients after corticosteroid therapy, reticular opacities deteriorated in six patients. In our patient, reticular opacities and PPFE‐like lesions remained unchanged despite treatment. IgG4‐RLD and lung fibrosis may have been accidentally coexisted in the present case. However, we believe that IgG4‐positive IP needs to be treated as a separate entity from conventional IgG4‐RLD because of differences in disease behaviour and responses to corticosteroid therapy. If his lung fibrosis progresses in the future, antifibrotic agents such as nintedanib may be effective as an additional therapy.

## CONFLICT OF INTEREST

None declared.

## ETHICS STATEMENT

The authors confirm that appropriate written informed consent of the patients was obtained for publication of this case report and accompanying images.

## Data Availability

The data that support the findings of this study are available on request from the corresponding author.
